# NAMING AND STORYTELLING IN SOCIAL ROBOT INTERACTIONS: A NARRATIVE ANALYSIS OF ENGAGEMENT AND PERSONAL CONNECTIONS

**DOI:** 10.1093/geroni/igae098.3554

**Published:** 2024-12-31

**Authors:** Clio Yuen Man Cheng, Vivian Weiqun Lou

**Affiliations:** The University of Hong Kong, Hong Kong, China (People’s Republic); The University of Hong Kong, Hong Kong, China (People’s Republic)

## Abstract

Social robots have emerged as promising tools with gerontology, offering the potential to enhance human-computer interaction and improve the well-being of older adults. This study employs narrative analysis approach to examine the impact of naming and storytelling in social robot interactions among older singletons. A total of 17 participants, aged between 60 to 73, engaged in 15-minute interaction sessions with a social robot (LOVOT), during which they provided a name for the robot and crafted a story around it. Through a comprehensive narrative analysis, this study uncovers the intricate dynamics of naming and storytelling in shaping older adults’ engagement and personal connections within social robot interactions. The analysis reveals that naming and storytelling serve as powerful vehicles for expressing expectations, personal experiences, and emotional connections. Participants drew upon their individual backgrounds, memories, and cultural references to create narratives that reflected their unique perspectives and desires. The narratives showcased the participants’ desires for intelligent and emotionally responsive robots while evoking nostalgia through connections to past experiences. Additionally, the narratives revealed a longing for companionship and emotional support, exemplified by associations of robot naming with deceased pets. Understanding the role of naming and storytelling in social robot interactions is crucial for designing robots that effectively cater to the needs and desires of older adults, ultimately enhancing their engagement and overall well-being. This study contributes to the field of human-robot interaction in gerontology by shedding light on the significance of personal narratives in fostering meaningful connections between older adults and social robots.

